# Obstetric A&E unit admission and hospitalization for obstetrical management during COVID-19 pandemic in a third-level hospital of southern Italy

**DOI:** 10.1007/s00404-021-06212-6

**Published:** 2021-08-29

**Authors:** Luigi Carbone, Antonio Raffone, Antonio Travaglino, Laura Sarno, Alessandro Conforti, Olimpia Gabrielli, Valentino De Vivo, Martina De Rosa, Sonia Migliorini, Gabriele Saccone, Mariavittoria Locci, Carlo Alviggi, Antonio Mollo, Maurizio Guida, Fulvio Zullo, Giuseppe Maria Maruotti

**Affiliations:** 1 Gynecology and Obstetrics Unit, Department of Neurosciences, Reproductive Sciences and Dentistry, School of Medicine, University of Naples Federico II, Via Sergio Pansini, 5, 80131 Naples, Italy; 2 Pathology Unit, Department of Advanced Biomedical Sciences, School of Medicine, University of Naples Federico II, Naples, Italy; 3 Department of Medicine, Surgery and Dentistry, Schola Medica Salernitana, University of Salerno, Baronissi, Salerno, Italy

**Keywords:** SARS-COV-2, Obstetric, Pregnant, Hospitalization, Admission, Infection

## Abstract

**Background:**

The COronaVIrus Disease 2019 (COVID-19) has spread in Italy since February 2020, inducing the government to call for lockdown of any activity, apart primary needs, during the months March–May 2020. During the lockdown, a reduction of admissions and hospitalizations for ischemic diseases was noticed. Purpose of this study was to observe if there has been the same reduction trend in Accident & Emergency (A&E) unit admissions also for obstetric-gynecological conditions.

**Methods:**

Medical records and electronic clinical databases were searched for all patients who were admitted to the obstetric A&E department or hospitalized at the Gynecology and Obstetrics Unit of University hospital of Naples Federico II, during the quarter March–May in the years 2019 and 2020. The mean ± standard deviation (SD) of monthly admission to the obstetric A&E department and hospitalization of the year 2020 was compared with that of the year 2019, using the unpaired *T* test with α error set to 0.05 and 95% confidence intervals (95% CI).

**Results:**

Admissions were 1483 in the year 2020 and 1786 in 2019. Of total, 1225 (37.5%) women were hospitalized: 583 in the year 2020, 642 in 2019. Mean ± SD of patients monthly admitted to our obstetric A&E department was 494 ± 33.7 in the year 2020, and 595.3 ± 30.9 in 2019, with a mean difference of − 101.3 (95% CI − 103.5 to − 99.1; *p* < 0.0001). Mean ± SD of patients monthly hospitalized to our department was 194 ± 19.1 in the year 2020, 213.7 ± 4.7 in 2019, with a mean difference of − 19.7 (95% CI − 23.8 to − 15.6; *p* < 0.0001).

**Conclusion:**

A significant decrease in the mean of monthly admissions and hospitalizations during the COVID-19 pandemic when compared to the previous year was found also for obstetric–gynecological conditions. Further studies are necessary to assess COVID-19 impact and to take the most appropriate countermeasures.

## Introduction

The Coronavirus disease 2019 (COVID-19) is a global public health emergency caused by the severe acute respiratory syndrome coronavirus 2 (SARS-CoV-2) [1, 2]. The first cases of COVID-19 in Italy dated on 30 January 2020, when two Chinese tourists were found positive in Rome. After, on 21 February, a cluster was registered in Codogno, in the North of the country. Soon, the infection spread around in the upper part of the nation, inducing the government to call for a strict lockdown on 8 March 2020. Since then, the COVID-19 pandemic has led to an extensive reorganization of health facilities to allow an adequate management of suspected or confirmed cases of SARS-COV-2 infection. Therefore, even gynecological and obstetrical units had to adapt their daily activities to offer care first to oncological or anyhow urgent cases both for pregnant and non-pregnant women, to reduce the crowding caused by the arrival of women for inpatient or outpatient visits [3, 4]. Being Italy one of the first European countries to be hit by the pandemic spread, national guidelines and papers were soon published to help hospitals speeding up the process of reorganization [5–12]. In this scenario, some hospitals including our Federico II University hospital of Naples, Italy, have been designated as regional hubs for the management of COVID-19 cases.

Since early during the pandemic, there was evidence of a drop of the number of people asking for emergency care throughout Italy, especially for ischemic heart disease [13, 14]. In regards to obstetric practice, our group showed how also invasive procedures for prenatal diagnosis dropped in the trimester of the lockdown (March–May 2020) in comparison with the same period of 2019 [15]. It has been hypothesized that the main cause for such behavior was the fear of going to hospitals due to the risk of contracting the infection, being in contact with other people or with potentially infected medical personnel [13, 14]. Furthermore, anxiety and behavioral changes have been soon observed in obstetric practice, possibly conducing to altered perception of the need of care during pregnancy [16, 17].

As for other diseases and conditions, obstetrics is characterized by urgency and emergency events which incidence should be more or less stable over the years. Therefore, this study was performed with the aim to evaluate if, similarly to what demonstrated for ischemic diseases, [13, 14] there has been a reduction in the admission rate to the obstetric Accident & Emergency (A&E) department and/or hospitalization rate at our facility, which is the biggest University hospital of South Italy, with around 2400 deliveries per year, during the period of the lockdown imposed by the government, compared to the previous year.

## Methods

### Study protocol

The study followed an a priori defined study protocol and was designed as a single-centre observational retrospective cohort study. It was reported according to the Strengthening the Reporting of Observational Studies in Epidemiology (STROBE) guidelines and checklist [18].

Medical records and electronic clinical databases were searched for all patients who were admitted to the obstetric A&E department or hospitalized at the Gynecology and Obstetrics Unit of the Department of Neurosciences, Reproductive Sciences and Dentistry, School of Medicine, University of Naples Federico II, Naples, Italy, during the quarter March–May in the years 2019 and 2020. The means of monthly admission to the obstetric A&E department and hospitalization were compared among the years.

### Study outcomes

Primary outcome was the difference in the mean of the patients monthly admitted to our obstetric A&E department during the quarter March–May between the years 2020 and 2019. Secondary outcome was the difference in the mean of the patients monthly hospitalized to our department during the quarter March–May between the years 2020 and 2019.

### Main analyses

The number of patients admitted to our obstetric A&E department and/or hospitalized was recorded for each month of the above-mentioned quarters, and the mean ± standard deviation (SD) of patients monthly admitted to our obstetric A&E department and/or hospitalized was separately calculated for the years 2020 and 2019.

Mean ± SD of patients monthly admitted to our obstetric A&E department and/or hospitalized of the year 2020 was compared with that of the year 2019, using the unpaired T test with α error set to 0.05 and 95% confidence intervals (95% CI).

Statistical analyses were performed using SPSS 19.0 package (SPSS Inc., Chicago, IL, USA).

### Additional analyses

Subgroup analyses assessing the difference in the mean ± SD of patients monthly admitted to our obstetric A&E department of the year 2020 with that of the year 2019 were performed based on the trimester of gestation (i.e. first, second and third trimester), the patient age (using 35 years old as cut-off), and the diagnosis at the admission.

## Results

### Characteristics of the study population

A total of 3269 patients admitted to our obstetric A&E department were included in the study. Admissions were 1483 in the year 2020 and 1786 in 2019. Of total, 1,225 (37.5%) women were hospitalized: 583 in the year 2020, and 642 in 2019.

2155 (66%) patients were under the age of 35 years old; 664 (20.3%) were in the first trimester of gestation, 526 (16.1%) in the second, and 1815 (55.5%) in the third; the remaining 264 women (8.1%) came for gynecological reasons.

Characteristics of the study population were reported in Table 1.

**Table 1:** Characteristics of the study population

Year	Age	Previous pregnancies	Previous miscarriage	Previous voluntary terminations of pregnancy	Previous ectopic pregnancies	Previous Cesarean sections	Previous stillborns	Previous IVF
2019	32.5 ± 7.3	0.3 ± 0.6	0.3 ± 0.7	0.05 ± 0.3	0.02 ± 0.1	0.01 ± 0.1	0.3 ± 0.7	0.02 ± 0.2
2020	31.7 ± 6.5	0.3 ± 0.7	0.3 ± 0.8	0.03 ± 0.2	0.01 ± 0.1	0	0.2 ± 0.6	0.01 ± 0.07
TOTAL	32.2 ± 7.2	0.3 ± 0.6	0.3 ± 0.7	0.04 ± 0.2	0.01 ± 0.1	0.01 ± 0.1	0.3 ± 0.6	0.02 ± 0.1

### Main analysis

Mean ± SD of patients monthly admitted to our obstetric A&E department was 494 ± 33.7 in the year 2020, and 595.3 ± 30.9 in 2019 (Table 2). Difference was − 101.3 (95% CI − 103.5 to − 99.1; *p* < 0.0001) (Table 3).

**Table 2:** Patients admitted to the obstetric A&E department and/or hospitalized

Item	2020									2019								
	Total			March		April		May		Total			March		April		May	
	*n*	%	Mean ± SD	*n*	%	*n*	%	*n*	%	*N*	%	Mean ± SD	*n*	%	*n*	%	*n*	%
Admissions to obstetric A&E department	1482	100	494 ± 33.7	448	30.2	506	34.2	528	35.6	1786	100	595.3 ± 30.9	552	30.9	622	34.8	612	34.3
Hospitalizations	583	39.3	194 ± 19.1	182	40.6	179	35.4	221	41.9	641	35.9	213.7 ± 4.7	217	39.3	217	34.9	207	33.8
*Age*																		
< 35	1028	69.3	342.3 ± 19.3	315	70.3	357	70.6	355	67.2	1128	63.2	376 ± 9.6	363	65.8	386	62.1	379	61.9
> 35	455	30.9	151.7 ± 16.4	133	29.7	149	29.4	173	32.8	658	36.8	219.3 ± 21.5	189	34.2	236	37.9	233	38.1
Miscarriage	48	3.2	16 ± 2.2	15	3.3	14	2.8	19	3.6	63	3.5	21 ± 2.2	18	3.3	23	3.7	22	3.6
Pelvic pain	276	18.6	92 ± 22.2	63	14.1	96	19.0	117	22.2	335	18.8	111.7 ± 18	89	16.1	133	21.4	113	18.5
Doppler abnormalities	2	0.1	0.7 ± 0.9	0	0	2	0.4	0	0	6	0.3	2 ± 1.4	1	0.2	4	0.6	1	0.2
Maternal anemia	3	0.2	1 ± 0.8	2	0.4	0	0	1	0.2	3	0.2	1 ± 0	1	0.2	1	0.2	1	0.2
IUGR	19	1.3	6.3 ± 3.3	4	0.9	4	0.8	11	2.1	22	1.2	7. ± 1.2	6	1.1	7	1.1	9	1.5
Uterine contractions	545	36.7	181.7 ± 17	159	35.5	186	36.8	200	37.9	472	26.4	157. ± 21.4	137	24.8	148	23.8	187	30.6
Hypertensive disorders	45	3.0	15 ± 5.9	23	5.1	13	2.6	9	1.7	27	1.5	9. ± 4.3	15	2.7	7	1.1	5	0.8
PROM/pPROM	110	7.4	36.7 ± 5.6	39	8.7	29	5.7	42	8.0	158	8.8	52.7 ± 2.6	49	8.9	55	8.8	54	8.8
Maternal weakness	10	0.7	3.3 ± 0.5	4	0.9	3	0.6	3	0.6	13	0.7	4.3 ± 1.7	2	0.4	6	1.0	5	0.8
Abnormal CTG pattern	9	0.6	3 ± 1.6	3	0.7	5	1.0	1	0.2	8	0.4	2.7 ± 1.9	4	0.7	4	0.6	0	0
Cough, dyspnea and chest pain	3	0.2	1 ± 0.8	1	0.2	2	0.4	0	0	13	0.7	4.3 ± 2.1	7	1.3	2	0.3	4	0.7
Hyperemesis	5	0.3	1.7 ± 0.4	2	0.4	2	0.4	1	0.2	18	1.0	6 ± 0.8	5	0.9	6	1.0	7	1.1
Hyperpyrexia	11	0.7	3. 7 ± 1.7	6	1.3	2	0.4	3	0.6	11	0.6	3.7 ± 1.7	2	0.4	3	0.5	6	1.0
Post-term pregnancy	9	0.6	3 ± 2.2	2	0.4	6	1.2	1	0.2	9	0.5	3 ± 2.2	5	0.9	0	0	4	0.7
Abnormal uterine bleeding	282	19.0	94 ± 6.5	88	19.6	103	20.4	91	17.2	394	22.1	131.3 ± 13.4	136	24.6	145	23.3	113	18.5
Cholestasis, hypertransaminasemia	11	0.7	3.3 ± 1.2	2	0.4	5	1.0	3	0.6	9	0.5	3 ± 1.6	5	0.9	1	0.2	3	0.5
Stillbirth	2	0.1	0.7 ± 0.9	2	0.4	0	0	0	0	4	0.2	1.3 ± 1.2	1	0.2	0	0	3	0.5
Amniotic fluid alterations	8	0.5	2. 7 ± 1.9	0	0	4	0.8	4	0.8	9	0.5	3 ± 1.4	4	0.7	1	0.2	4	0.7
Reduced fetal movement	28	1.9	9.3 ± 0.9	10	2.2	10	2.0	8	1.5	41	2.3	13.7 ± 2.6	10	1.8	16	2.6	15	2.5
Extrauterine pregnancy	15	1.0	5 ± 0.8	5	1.1	4	0.8	6	1.1	17	1.0	5.7 ± 1.2	6	1.1	4	0.6	7	1.1
Abdominal pain	15	1.0	5 ± 2.2	7	1.6	6	1.2	2	0.4	64	3.6	21.3 ± 5.2	25	4.5	14	2.3	25	4.1
Post-operative complications	3	0.2	1 ± 0.8	2	0.4	1	0.2	0	0	7	0.4	2.3 ± 1.6	0	0	3	0.5	4	0.7
Bartholin's cyst	7	0.5	2.3 ± 1.7	4	0.9	3	0.6	0	0	2	0.1	1.3 ± 0.5	1	0.2	2	0.3	1	0.2
Other diseases	15	1.1	5 ± 0.8	4	0.9	6	1.2	5	0.9	66	3.7	22 ± 5.7	19	3.4	30	4.8	17	2.8
Polypathology	2	0.1	0.7 ± 0.5	1	0.2	0	0	1	0.2	15	0.8	5 ± 1.4	4	0.7	7	1.1	4	.7
*Trimester*																		
First	280	18.9	93.3 ± 3.7	89	19.9	98	19.4	93	17.6	384	21.5	128 ± 7	127	23.0	137	22.0	120	19.6
Second	187	12.6	62.3 ± 10.5	53	11.8	57	11.3	77	14.6	339	19.0	113 ± 8.2	103	18.7	113	18.2	123	20.1
Third	935	63.0	311.3 ± 26	276	61.6	320	63.2	338	64.0	880	49.3	293.3 ± 21.4	263	47.6	308	49.5	309	50.5

**Table 3:** Difference in means ± SD of patients monthly admitted to the obstetric A&E department and/or hospitalized between the years 2020–2019

Item	2020–2019		
	Difference	95% CI	*P*
Admissions to A&E department	− 101.3	− 103.5 to − 99.1	< 0.0001
Hospitalizations	− 19.7	− 23.8 to − 15.6	< 0.0001
Age < 35 years	− 33.7	− 34.973 to − 32.427	< 0.0001
Age > 35 years	− 67.6	− 69.945 to − 65.255	< 0.0001
Miscarriage	− 5	− 10.522 to 0.522	0.0755
Pelvic pain	− 19.7	− 22.895 to − 16.505	< 0.0001
Doppler abnormalities	− 1.3	− 3.957 to 1.357	0.2763
Maternal anemia	0	− 1.282 to 1.282	1
IUGR	− 0.7	− 6.456 to 5.056	0.8070
Uterine contractions	24.7	22.332 to 27.068	< 0.0001
Hypertensive disorders	6	3.397 to 8.603	< 0.0001
PROM/pPROM	− 16	− 17.003 to − 14.997	< 0.0001
Maternal weakness	− 1	− 2.160 to 0.160	0.0874
Abnormal CTG pattern	0.3	− 1.509 to 2.109	0.7286
Cough, dyspnea and chest pain	− 3.3	− 6.003 to − 0.597	0.0202
Hyperemesis	− 4.3	− 5.079 to − 3.521	< 0.0001
Hyperpyrexia	0	− 1.512 to 1.512	1
Post-term pregnancy	0	− 2.199 to 2.199	1
Abnormal uterine bleeding	− 37.3	− 39.087 to − 35.513	< 0.0001
Cholestasis, hypertransaminasemia	0.3	− 1.014 to 1.614	0.6374
Stillbirth	− 0.6	− 3.323 to 2.123	0.5738
Amniotic fluid alterations	− 0.3	− 2.011 to 1.411	0.7139
Reduced fetal movements	− 4.4	− 5.422 to − 3.378	< 0.0001
Ectopic pregnancy	− 0.7	− 1.447 to 0.047	0.0653
Abdominal pain	− 16.3	− 19.040 to − 13.560	< 0.0001
Post-operative complications	− 1.3	− 3.595 to 0.995	0.2278
Bartholin's cyst	1	− 2.005 to 4.005	0.4572
Other diseases	− 17	− 22.122 to − 11.878	< 0.0001
Polypathology	− 4.3	− 6.480 to − 2.120	0.0008
Trimester			
First	− 34.7	− 35.602 to − 33.798	< 0.0001
Second	− 50.7	− 52.326 to − 49.074	< 0.0001
Third	18	15.796 to 20.204	< 0.0001

Mean ± SD of patients monthly hospitalized to our department was 194 ± 19.1 in the year 2020, and 213.7 ± 4.7 in 2019 (Table 2). Difference was − 19.7 (95% CI − 23.8 to − 15.6; *p* < 0.0001) (Table 3).

### Additional analyses

#### Age

Mean ± SD of patients under the age of 35 monthly admitted to our obstetric A&E department was 342.3 ± 19.3 in the year 2020, and 376 ± 9.6 in 2019 (Table 2). Difference was − 33.7 (95% CI − 34.973 to − 32.427.1; *p* < 0.0001) (Table 3).

Mean ± SD of patients over the age of 35 monthly hospitalized to our obstetric A&E department was 151.7 ± 16.4 in the year 2020, and 219.3 ± 21.5 in 2019 (Table 2). Difference was − 67.6 (95% CI − 69.945 to − 65.255; *p* < 0.0001) (Table 3).

#### Trimester of gestation

Mean ± SD of patients admitted in the first trimester of gestation was 93.3 ± 3.7 in the year 2020, and 128 ± 7 in 2019 (Table 2). Difference was − 34.7 (95% CI − 35.602 to − 33.798; *p* < 0.0001) (Table 3).

Mean ± SD of patients admitted in the second trimester of gestation was 62.3 ± 10.5 in the year 2020, and 113 ± 8.2 in 2019 (Table 2). Difference was − 50.7 (95% CI − 52.326 to − 49.074; *p* < 0.0001) (Table 3).

Mean ± SD of patients admitted in the third trimester of gestation was 311.3 ± 26 in the year 2020, and 293.3 ± 21.4 in 2019 (Table 2). Difference was + 18 (95% CI 15.796–20.204; *p* < 0.0001) (Table 3).

#### Diagnosis at the admission

Means ± SD of patients monthly admitted to our obstetric A&E department stratified by diagnosis at the admission with difference between the years 2020 and 2019 were reported in Tables 2 and 3, respectively.

Differences were found significant for pelvic pain, uterine contractions, hypertensive disorders, premature rupture of membranes (PROM)/ preterm premature rupture of membranes (pPROM), hyperemesis, abnormal uterine bleeding, reduced fetal movement, abdominal pain, other diseases and polypathology (Table 3).

## Discussion

This study showed a significant decrease in the mean of monthly admissions and hospitalizations during the COVID-19 pandemic when compared to the previous year.

Other nine studies analyzed the issue of the impact of COVID-19 pandemic on the admissions to the A&E unit for obstetrical or gynecological conditions. Two of them were performed in the United States, [19, 20] two in Israel, [21, 22] one in France, [23] one in India, [24] and three in Italy (notably from regions different from ours) [25–27] (Table 4). Only Goyal et al. [24] performed a prospective analysis, while all the others were retrospective studies. The biggest cohorts were from Abel et al. [19] and Athiel et al. [23] with overall 11,788 and 39,690 patients seen in the period considered, respectively, although it’s worth mentioning that the French study was multicentric. Looking to the period of analysis considered by the various studies, we noticed that Abel et al. [19] compared 3 periods: pre-pandemic, early pandemic and late pandemic; Athiel et al. [23], Goyal et al. [24], Spurlin et al. [20], and Grandi et al. [26] considered the period preceding the pandemic *versus* the first lockdown period; Kugelman et al. [22] evaluated the month following the pandemic declaration, Salsi et al. [25] assessed the month of March (therefore, immediately before and during the lockdown), and Dell’Utri et al. [27] comprised almost 4 months, from the case of the first COVID-19 affected Italian patient to after the end of the strict lockdown *versus* the same period of the year 2019; Meyer et al. [21] included two months in their analysis (February and March, which according to what mentioned by Kugelman et al. [22] regarding the first Israeli patient affected means before and immediately after the recognition of SARS-COV-2 diffusion in Israel), comparing to the previous year.

**Table 4:** Studies evaluating the impact of COVID-19 pandemic on obstetrical A&H unit admissions in the Literature

Authors, Year	Study location	Study design	Period considered	Patients admitted to the A&E units (cases vs controls)
Abel et al. 2020 [19]	San Francisco, USA	Retrospective case–control	March 4–May 19, 2020 vs January 1–March 3, 2020	11,788 (4903 vs.6885)
Athiel et al. 2020 [23]	France	Retrospective case–control	March–May 2020 vs 2019	39,690 (14,708 vs. 24,982)
Carbone et al. 2020 [7]	Naples, Italy	Retrospective case–control	March–May 2020 vs 2019	3269 (1483 vs. 1786)
Dell’Utri et al. 2020 [27]	Milan, Italy	Retrospective case–control	February–June 2020 vs 2019	9291 (3647 vs. 5644)
Goyal et al. 2020 [24]	Jodhpur, India	Prospective, case–control	April 1, 2020, to August 31, 2020 vs. October 1, 2019, to February 29, 2020	1749 (633 vs 1116)
Grandi et al. 2020 [26]	Modena-Sassari-Cagliari, Italy	Retrospective case–control	March 11–April 9, 2020 vs. November 1 to 30, 2019	691 (209 vs. 482)
Kugelman et al. 2020 [22]	Haifa, Istrael	Retrospective case–control	March 15, 2020–April 12, 2020 vs March 15, 2019–April 12, 2019	942 (398 vs. 544)
Meyer et al. 2020 [21]	Israel	Retrospective case–control	February–March 2020 vs 2019	7964 (3897 vs 4067)
Salsi et al. 2020 [25]	Bologna, Italy	Retrospective case–control	March 2020 vs 2019	1456 (484 vs. 972)
Spurlin et al. 2020 [20]	New York City, USA	Retrospective case–control	February 1 to March 15 vs. March 16 to April 15	354 (79 vs 275)

Regarding the assessed outcomes, all the studies showed the number of A&E admissions for obstetrical or gynecological issues, but only two of them reported the number of patients by weeks and none did it by months. In particular, Dell’Utri et al. [27] showed weekly numbers for 13 weeks, and Meyer et al. [21] for 8 weeks.

In our study, when we analyzed the data according to the age, we did not find a difference in mean age at admission compared with the previous year. In particular, we found a significant decrease in admissions for both under and over the cut-off age of 35. In accordance, Meyer et al. [21], Kugelman et al. [22], and Grandi et al. [26] reported mean age at admission, with no statistically significant differences among groups.

Looking to the stage of pregnancy, patients in their first and second trimester were found to seek less frequently medical care. Oppositely, the rate of admission during the last trimester was increased compared to the past. Salsi et al. [2, 5] divided the pregnant patients in before and after 16 weeks, while Dell’Utri et al., considered three subgroups but not properly dividing by trimesters as we did; both studies found a reduction in the admission rates irrespectively of gestational ages. On the contrary, Grandi et al. [26] in a multicenter Italian study from three university hospitals, observed an increase in the number of triages during the lockdown for first trimester of pregnancy patients. However, their analysis compared the period of the lockdown to November 2019, with possible biases given by seasonal differences which they already acknowledged.

Regarding the number of deliveries, as showed in another study from our group, [15], it appeared increased in the trimester March–May 2020 compared to the same trimester 2019, in accordance to what described by Dell’Utri et al. [27]. As an explanation, patients about to give birth preferred to come to our facility, known to be equipped as regional hub with dedicated routes for COVID-19 patients, instead of going to smaller hospitals with a supposed increased risk of infection. On the other hand, Goyal et al. [24] observed a reduction of deliveries and Meyer et al. [21] did not find any difference in the weekly numbers.

Interestingly, reasons for admission as pelvic pain and abdominal pain were reduced compared to the 2019. Pelvic pain or abdominal pain represent generic reasons for admission to the A&E obstetric unit, and very often are used as excuses to have a rapid check on pregnancy status when something trivial happens and worries the pregnant women. In Italy, A&E unit is a free of charge National Health Service (NHS) task, which is usually overwhelmed by non-urgent request for medical care. Our results prove that during the pandemic less people sought medical care when it was not important.

On the other hand, in 2020, women with referred uterine contractions were more frequently admitted to the A&E obstetric unit. An explanation for this phenomenon, in accordance to what above-mentioned, it could be that hospital care was sought only when with impending labor symptoms, and not with weaker pains. Kugelman et al. [22] in accordance to us, noticed an increase in the number of women coming to hospital to be admitted to the labor ward; oppositely, Salsi et al. [25] found a reduction in this reason for admission.

Hypertensive disorders appeared increased in 2020 compared to the 2019. It could be assumed that the lifestyle imposed by the lockdown would have increased the risk for the development of such complications. Indeed, less physical activity, more home rest, and a related increased maternal weight, in addition to stress and anxiety, could constitute possible determinants for the increase in blood pressure. Accordingly, Salsi et al. [25] observed how hypertensive disorders have been an increased reason to seek for medical assistance, although their data are not statistically significant. On the other hand, Dell’Utri et al. [27] showed unchanged rates of admission for hypertensive disorders.

Leakage of amniotic fluid (PROM or pPROM) was found as a reduced cause to seek for A&E unit assistance in 2020 compared to the past, maybe due to the fact that many cases in the past were misunderstanding this event, running to the hospital when vaginal discharge or urine leakage occurred. Salsi et al. [25] results agree with ours. Differently, Kugelman et al. [22] observed an increased rate of A&E admissions for such issue compared to 2019.

Cough, dyspnea and chest pain cases seemed to be reduced in 2020 compared to the 2019. Being a reason strictly correlated to the symptoms of COVID-19, it is possible that the fear of being infected led to search first for the proper general practitioner, who was advocated by national authorities as the first provider to call in case of symptoms, and who could have resolved the case without need to send the patient to the hospital, being a non-obstetric reason. Worth to be mentioned is the application, in this subset of patients, of lung ultrasound to provide early diagnosis of lung involvement, which could reduce the request for X-ray application in the management of COVID-19 obstetric patients [28, 29]. Along the same lines, there was no difference in admission rate for fever comparing 2020 with the previous year.

Hyperemesis was markedly reduced in 2020 compared to the past. Being almost entirely a first trimester symptom, and having women in first trimester asked less frequently for medical care, it is plausible that as pelvic pain, such reason is just overrepresented by patients in their initial stage of pregnancy due to general anxiety for the outcome of the fetus, resulting in more medical visits than effectively needed, which event did not happen in 2020 due to the fear of hospitals. Only Salsi et al. [25] looked to this reason for admission, showing as well a reduction.

Similarly, reduced fetal movements, typically a third trimester symptom, were noticed to be less frequently claimed as reasons for admission to the A&E unit. Non-significant drops were observed by Kugelman et al. [22] and Salsi et al. [25].

Abnormal uterine bleeding, irrespective of the trimesters, was reduced as well in 2020 compared to the past. Abel et al. [19] and Dell’Utri et al. [27] found the same. A possible reason could be that, as for the increase in hypertensive disorders, a more restful lifestyle determined less pelvic cramps (and pain, as above showed) and so less risk of amnio-chorial detachment or true placental abruption. On the other hand, Kugelman et al. [22] observed a non-significant reduction, while Salsi et al. [25] showed a significant reduction for < 16 weeks pregnant women, but not for > 16 weeks patients.

Furthermore, we included all the reasons for admission other than those specified within the category “other diseases”, and as “polypathology” the sum of more than one condition. Both outcomes showed a reduction in comparison to the previous year, once again demonstrating how miscellaneous reasons were often used to gain access to rapid and free of charge visits, and also that the period of lockdown has probably influenced the incidence, or even the recognition, of coexistence of pathologic conditions, as well as the diagnosis of myocardial infarction were demonstrated to be reduced in the same time frame [1]

Khalil et al. [30] demonstrated how there was a drop in the number of obstetric triages at St George’s University Hospital, London, UK, with a parallel reduction of hospital births; in contrast, the number of prenatal bookings did not differ. Khalil et al. [31] reported also that the number of stillbirths increased during the pandemic. In this regard, despite a small fall of such cases has been noticed at our hospital, the results were not statistically significant.

A strength of our analysis comes from the high number of observations and also the wide variety of diagnosis considered at admission. On the other side, the retrospective nature of the study represents a limitation. Multicenter studies, with common protocols for admissions and for the definitions of diagnosis at admission, could better acknowledge the impact of COVID-19 on the rate of presentations to the obstetrical A&H unit. Reporting monthly data would also easily allow the comparisons of studies and pooling data. As another limitation, we were not able yet to measure the impact of such a reduction of A&E admissions on the outcomes of pregnancy. However, people should not underestimate their symptoms as well as maybe they should not overestimated in the past. COVID-19 is not only a problem for infected people but also plays a role also in reducing the NHS capacity of providing assistance to the other people ill for different reasons. Therefore, it appears of outstanding importance to ensure that hospitals and medical personnel are sought in really urgent cases, but also that fear of contracting the infection would not prompt patients to avoid medical care. This goal should be quickly reached, providing adequate information to the population on how to follow strict rules to seek hospital assistance, if and only a problem arises. Vaccines against SARS-COV-2 have now been produced and released; national societies recommend their use in pregnant women, [32, 33] although preliminary reports show patients’ fear for eventual adverse events and safety concerns for the fetus [34, 35]. Indeed, their diffusion will hopefully reduce the burden of the pandemic and consequently the impact on healthcare systems. However, the return to previous habits should take into consideration what happened until now, to reduce in the future the eventually useless overcrowding of A&E units.

## Conclusion

Admissions and hospitalizations have been reduced during the lockdown trimester in Naples university hospital, in comparison to the same period of 2019, although the number of deliveries slightly increased. These findings seem due to the fear of contracting the infection in hospitals. Moreover, as in Italy A&E unit is a free of charge NHS task, the decrease in admissions seems indicate an improper use of A&E unit by patients in non-emergency period. Non-urgent requests for medical care seem to underlie such decrease. On the other hand, the fear of contracting the infection would not prompt patients to avoid medical care. It appears of outstanding importance to provide adequate information to the population on how to seek hospital assistance. Further studies are necessary to assess COVID-19 impact to take the most appropriate countermeasures.
